# Celastrol Blocks Interleukin-6 Gene Expression via Downregulation of NF-κB in Prostate Carcinoma Cells

**DOI:** 10.1371/journal.pone.0093151

**Published:** 2014-03-24

**Authors:** Kun-Chun Chiang, Ke-Hung Tsui, Li-Chuan Chung, Chun-Nan Yeh, Wen-Tsung Chen, Phei-Lang Chang, Horng-Heng Juang

**Affiliations:** 1 Department of General Surgery, Chang Gung Memorial Hospital, Keelung, Taiwan, ROC; 2 Department of Urology, Chang Gung Memorial Hospital, Kwei-Shan, Tao-Yuan, Taiwan, ROC; 3 Department of Anatomy, School of Medicine, Chang Gung University, Kwei-Shan, Tao-Yuan, Taiwan, ROC; 4 Department of General Surgery, Chang Gung Memorial Hospital, Kwei-Shan, Tao-Yuan, Taiwan, ROC; 5 National Kaohsiung University of Hospitality and Tourism, Hsiao-Kang, Kaohsiung Taiwan, ROC; Innsbruck Medical University, Austria

## Abstract

Interleukin-6 (IL-6), a multifunctional cytokine, contributes to proliferation or differentiation of prostate carcinoma cells in a highly cell type-specific manner. Celastrol (3-hydroxy-24-nor-2oxo-1(10),3,5,7-friedelatetrane-29-oic acid), also named as tripterine, is extracted from root of Chinese traditional herb *Tripterygiumwilfordii* Hook f with potent anti-inflammatory and anti-cancer activities. In this study, we evaluated the molecular mechanisms of celastrol on cell proliferation and IL-6 gene expression in prostate carcinoma cells. ^3^H-thymidine incorporation and flow cytometric analysis indicated that celastrol treatments arrested the cell cycle at the G0/G1 phase, thus attenuating cell proliferation in prostate carcinoma PC-3 cells; moreover, celastrol induced cell apoptosis at higher dosage. Knockdown of IL-6 attenuated the anti-proliferative effect of celastrol on PC-3 cells. Results from ELISA and 5’-deletion transient gene expression assays indicated that celastrol treatment decreased IL-6 secretion and gene expression, and this effect is dependent on the NF-κB response element within IL-6 promoter area since mutation of the NF-κB response element from AAATGTCCCATTTTCCC to AAATGTTACATTTTCCC by site-directed mutagenesis abolished the inhibition of celastrol on the IL-6 promoter activity. Celastrol also attenuated the activation of PMA and TNFα on the gene expression and secretion of IL-6 in PC-3 cells. Immunoblot assays revealed that celastrol treatment downregulated the expressions of IKKα, p50 and p65, supporting the 5’-deletion transient gene expression assay result that celastrol blocked IL-6 expression through the NF-κB pathway in PC-3 cells. For the first time, our results concluded that celastrol attenuates PC-3 cell proliferation via downregulation of IL-6 gene expression through the NF-κB-dependent pathway.

## Introduction

Prostate cancer is the second most common solid tumor for men in United States with 28,170 patients dying of this disease in 2012 [1]. Although the early diagnosis is more feasible due to the recent improvement of prostate-specific antigen (PSA) measurement, which improves the overall survival of prostate cancer patients, however, for the 15% of prostate cancer patients categorized as high-risk prostate cancer, 30–60% of them at around 10 years would eventually have metastasis with 10–25% patients dying of metastasis. [2], [3]. Currently, no consensus on the optimal management of high-risk patients is available. Multimodal approaches seem to have better outcome than the single-modality treatment. Under this bleak background, development of a new therapeutic regimen to treat prostate cancer should be prioritized.

Recently, to discover new potent anti-tumor compounds with less-toxic characteristics from Chinese natural medicine is getting popular. Among these compounds, celastrol (or tripterine), a quinine methidetriterpenoid, is derived from the root of Trypterigiumwilfordii (also known as Thunder of God Vine) [4], [5]. Celastrol has been implicated with potent anti-inflammation and anti-tumor effects in ample studies. So far, celastrol has been shown to have beneficial effects on a variety of cancers *in vitro* and *in vivo*, such as breast cancer, melanoma, squamous cell cancer, and prostate cancer [6]–[9].

Interleukin 6 (IL-6) is a glycoprotein consisting of 184 amino acids. IL-6 is first identified as a T-cell-derived regulation factor controlling B cell differentiation. IL-6 is now to be known to have multi-functions in a variety of cells and tissues [10]. Since the cloning of IL-6 cDNA, IL-6 has been proved to be produced in varied kinds of cells, including cancer cells, in addition to T- cell. IL-6 has been shown to involve in a number of important biological activities, including immune modulation, pro-inflammation, oncogenesis, and pro- or de- differentiation, in a highly cell- or tissue- specific way. In terms of prostate cancer, IL-6 has been demonstrated to be able to induce androgen receptor expression and promote tumor progression [11], [12], thus deemed as a growth factor for most prostate cancer cells *in vitro*. Some transcriptional factors have been reported to involve in the modulation of IL-6 gene expression and have binding sites within the IL-6 promoter area, including AP-1, cAMP, NF-κB, etc [13].

Previously, celastrol has been shown to possess anti-growth effect on prostate cancer *in vitro* and *in vivo* [8], [14], [15]. In this current study, we aimed to investigate the underlying mechanism whereby celastrol inhibits prostate cancer growth. We found for the first time that celastrol inhibited IL-6 secretion and expression in PC-3 cells, one kind of prostate cancer cells, which partly contributed to the anti-proliferative effect of celastrol on PC-3 cell. The secretion and expression of IL-6 in DU-145 cells was also repressed by celastrol. Further, we also provided the first laboratory evidence that celastrol repressed IL-6 secretion and expression in a NF-κB-dependent pathway in PC-3 cells.

## Materials and Methods

### Materials, cell lines, and cell culture

PC-3 and DU145 cells were obtained and maintained as described previously [16]. Celastrol and phorbol 12-myristate 13-acetate (PMA) were purchased from Sigma (St. Louis, MO). The stock celastrol (10 mM) was dissolved in DMSO. TNFα was purchase from PeproTech (Rebovot, Israel). The culture media were purchased from Life Technologies (Rockville, MD), and fetal calf serum (FCS) was from the HyClone (Logan, Utah). PC-3 and DU145 cells were cultured in RPMI 1640 medium with 10% FCS. The control groups in this experiment were treated with DMSO.

### Cell proliferation assay

Cell proliferation in response to celastrol was measured using a ^3^H-thymidine incorporation assay as previously described [16].

### Flow cytometry

Cells were serum starved for 24 hours and then cultured in RPMI 1640 medium with 10% FCS and with or without different concentrations of celastrol for another 48 hours. The cells were collected and stained with propidium iodide. Cell cycle analysis was performed using the FACS-Calibur cytometer and CellQuestPro software (BD Biosciences, San Jose, CA); the data were analyzed using ModFit LT Mac 3.0 software as previously described [17].

### Immunoblot Assay

Cells were incubated in the RPMI-1640 medium with 10% FCS and different treatments for a period of 24 hours. The nuclear and cytoplasmic fractions were extracted by NE-PER Nuclear and Cytoplasmic Extraction Reagent Kit (Thermo Scientific, Rockford, IL). Equal quantities of cell extract were loaded onto a 10% sodium dodecyl sulfate polyacrylamide (SDS) gel and analyzed by the electrochemiluminescent detection system. The blotting membranes were probed with 1∶1000 diluted IκB kinase α (IKKα) antiserum, 1∶1000 NFκBp50 antiserum, 1∶1000 diluted NFκBp65 antiserum (Merck Millipore, Darmstadt, Germany), 1∶1000 diluted PARP (Cell Signaling, Danvers, MA), 1∶200 diluted NFκB-inducing kinase (NIK) antiserum, 1∶1000 diluted IκB antiserum, 1∶200 diluted Lamin B antiserum, or 1∶3000 diluted β-actin antiserum (Santa Cruz Biotechnology, Santa Cruz, CA). The intensity of different bands was recorded and analyzed by GeneTools of ChemiGenius (Syngene, Cambridge, UK).

### Tunnel assay

After one day of treatment, cellular DNA was stained by TumorTACS in Situ Apoptosis Detection kit (TREVIGEN #4815-30-K). The assay was performed according to the manufacturer’s instructions.

### Knockdown of IL-6

The pSMc2 retroviral vectors containing the IL-6 short hairpin RNA (shRNA; V2HS-111640) and the GFP shRNA (RHS1764-9394112) were purchased from Open Biosystems (Huntsville, AL). The IL-6 shRNA and GFP shRNA vectors were introduced into PC-3 cells by electroporation using a singles 70-msec pulse of 180 V, and the transfections were selected using 2 μg/ml puromycin dihydrochloride. The IL-6-knockdown PC-3 cells were designated PC-IL6si cells and GFP-knockdown PC-3 cells were designated PC-COLsi cells as described previously [18].

### ELISA of IL-6

The IL-6 protein level in the culture medium was measured by ELISA (cat. No. 2107; Bio Scientific Corporation, Austin, TX). The relative mass of the IL6 protein present in each sample was determined based on the total protein concentration of the whole cell extract as described previously [19].

### Report Vector Constructs

The MMTV reporter vector was constructed as previously described [20]. The NF-κB reporter vector was purchase from Clontech (Moutain View, CA). The DNA fragment containing the enhancer/promoter of human IL-6 was isolated from the BAC clone (RPI11-240H8) and the reporter vectors containing the different fragments of 5’-flanking region of human IL-6 gene and mutant NF-κB response element were constructed as previously described [21].

### Luciferase and β-Galactosidasea assay

Cells were seeded onto 24-well plates at 1×10^4^ cells/well 1 day prior to transfection. The cells were transiently transfected using TransFast transfection reagent as described previously [19]. The media containing the liposome-DNA complex was removed and replaced with RPMI 1640 medium with 10% FCS for overnight. The media were replaced with RPMI 1640 medium with 10% FCS with different concentrations of celastrol, TNFα, or PMA as indicated for further 24 hours. Cells were harvested for activities of luciferase and β-galactosidase as described by the manufacturer instructions (Promega Bioscience, Madison, WI,).

### Statistical analysis

Results are expressed as the mean ± S.E. of at least three independent replication of each experiment. Statistical significance was determined by one way ANOVA and pair-*t* test analysis with program of SigmaStat for Window version 2.03 (SPSS Inc, Chicago, IL).

## Results

**Figure 1:**
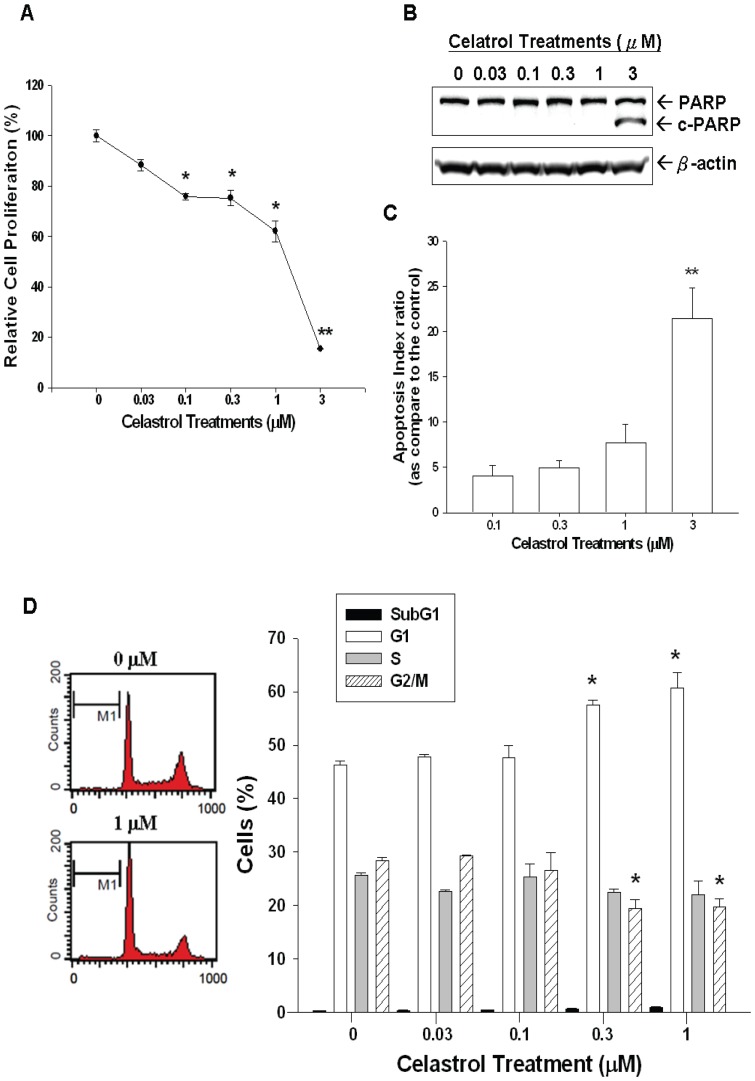
Celastrol inhibits PC-3 cell growth through cell cycle arrest at G0/G1 and apoptosis induction. (A) PC-3 cells were treated with indicated concentrations of celastrol for 48 hours and the cell proliferation was determined by the H^3^-thymidine incorporation. (B) PC-3 cells were treated with indicated concentrations of celastrol for48 hours. Cells were lysed and expressions of PARP, cleaved PARP (c-PARP) were determined by immunoblotting assay. (C) After one day of treatment, the apoptotic index of PC-3 cells treated with different concentrations of celastrol was calculated. Each value is a mean ± SE of 3 determinations. (D) PC-3 cells were serum starved for 24 hours and then were treated with 0 – 1 μM of celastrol as indicated for 48 hours. The cells were stained with PI, and the cell cycle distribution was analyzed by flow cytometry. Each box represents the mean ± SE (n = 6). (* *p*<0.05; **p<0.01).

Cell proliferation in the PC-3 cells was measured by ^3^H-thymidine incorporation assay. Results indicated cell proliferation decreased 37% when cells were treated with 1 μM of celastrol and 80% cell proliferation inhibition was observed as treated by 3 μM celastrol for 48 hours (Figure 1A). Immunoblot assay revealed that 3 μM of celastrol induced cleaved form of PARP (c-PARP) expression in PC-3 cells, indicating apoptosis induction (Figure 1B). To confirm apoptosis induction by high dose of celastrol, we further conducted tunnel assay. As shown in Figure 1C, after one day of treatment, 3 μM celastrol induced obvious apoptosis in PC-3 cells with an apoptosis index ratio of 21±3.2. Therefore, we used the proapoptosis (≤ 1 μM) dosage of celastrol for further studies below. Results from flow cytometric analysis revealed that celastrol induced cell cycle arrest at G0/G1 phase in PC-3 cells dose-dependently after 48 hours treatment with 1 μM of celastrol inducing 16% increase in G0/G1 phase cells together with a decrease in S phase cells (Figure 1D).

**Figure 2:**
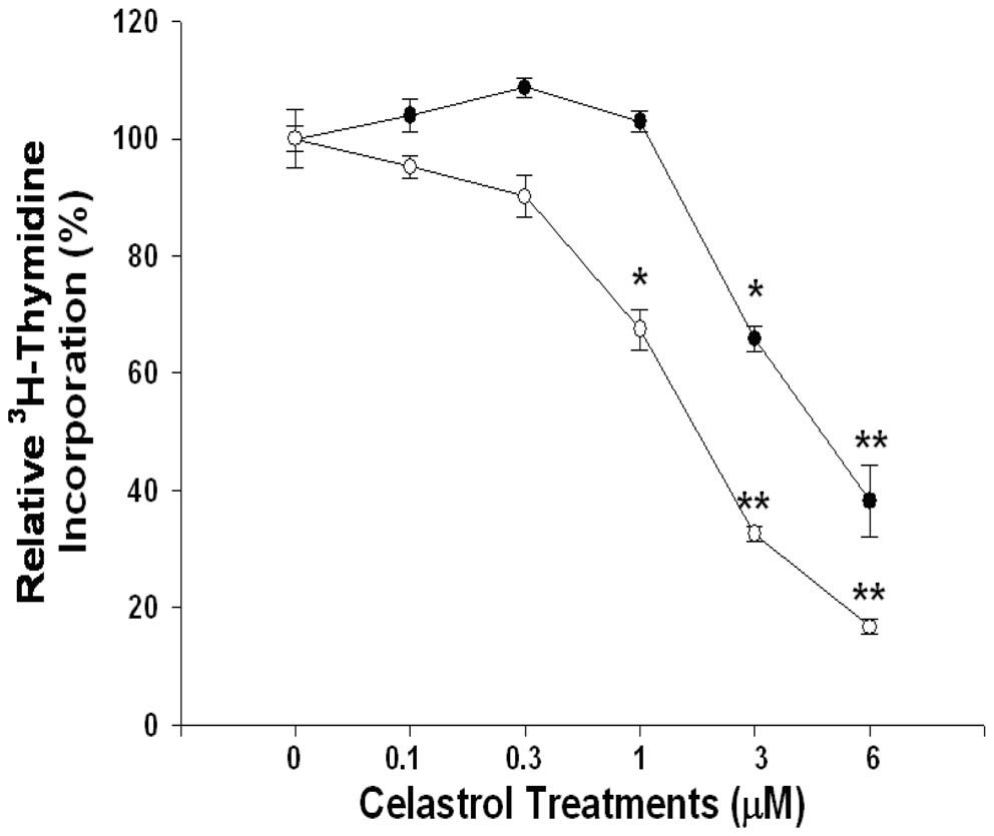
Knockdown of interleukin-6 attenuates the growth-inhibitorty effect ofcelastrol onPC-3 cells. PC-COLsi cells (open circle) and PC-IL6si cells (close circle) were treated with various concentrations of celastrol, as indicated, for 48 hours. The cell proliferation was determined by the ^3^H-thymidine incorporation. Data are presented as mean percentage ± SE (n = 6). (* *p*<0.05; **p<0.01).

*In vitro* studies revealed that knockdown of IL-6 significantly (*P* = 0.0217) attenuated the blocking effect of celastrol on cell proliferation in PC-3 cells as determined by the ^3^H-thymidine incorporation assay. As shown in figure 2, 48 hours 1, 3, and 6 μM celastrol treatments induced 32.6%, 77.4%, and 83.3% growth inhibition, respectively, in PC-COLsi cells. In the contrast, the same dosage of celastrol treatments repressed PC-IL6si cells growth by only 0%, 44.1%, and 61.8%, respectively (Figure 2).

**Figure 3:**
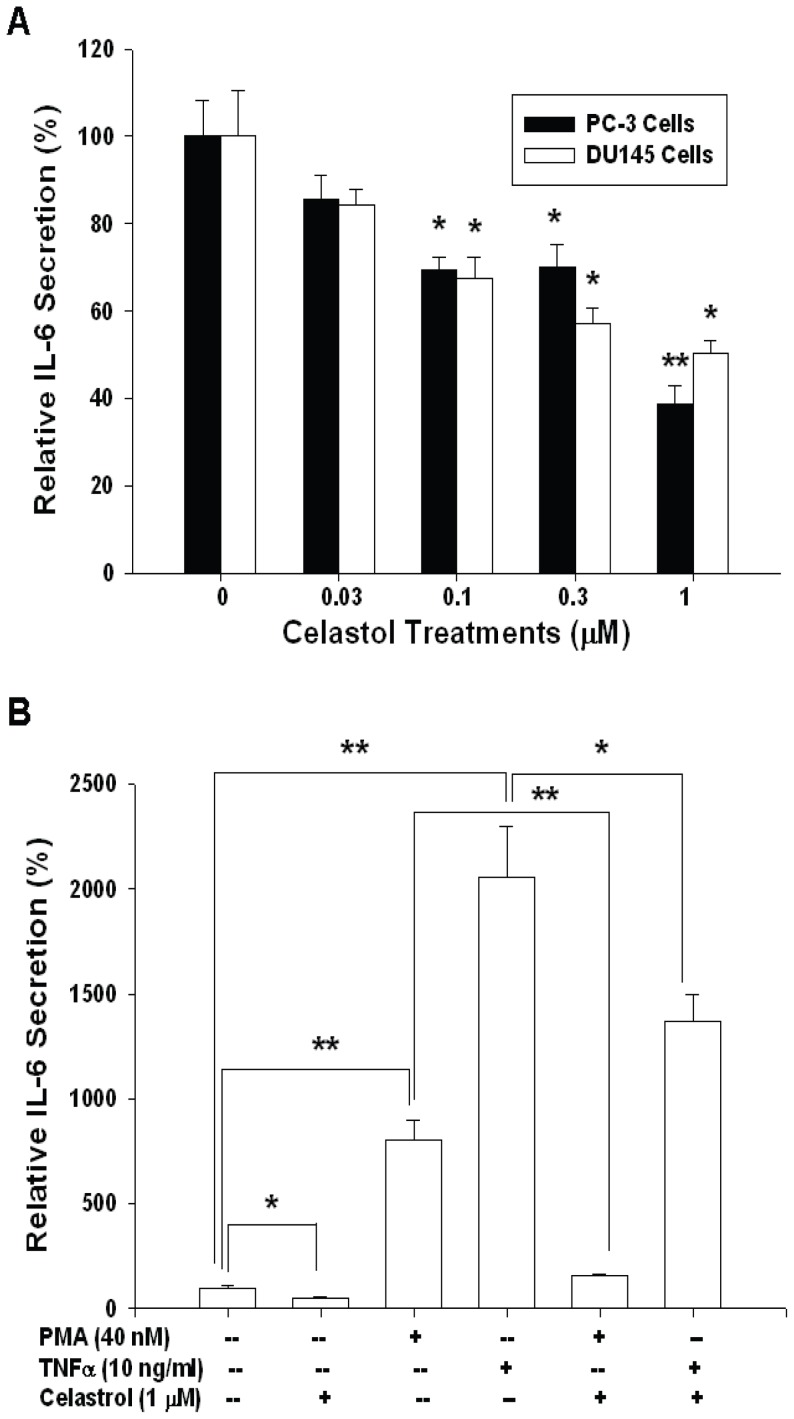
Celastrol Modulates interleukin-6 secretion in PC-3 cells. (A) PC-3 (black bars) and DU145 (white bars) cells were treated with various concentrations of celastrol, as indicated, for 24 hours. (B) PC-3 cells were treated with 1 μM celastrol, 40 nM PMA, and/or 10 ng/ml TNFα for 24 hours. IL-6 levels in the conditioned media were determined by ELISA. Data are expression as mean percentage stimulation ± SE of 6 preparation induced by different treatments relative to the control solvent treatment. (*p<0.05; **p<0.01).

Results from ELISA indicated that celastrol blocked IL-6 secretion of PC-3 and DU145 cells in a dosage-dependent manner. 1 μM celastrol treatment blocked 62% of IL-6 secretion (Figure 3A). Further ELISA revealed that PMA (40 nM) and TNFα (10 ng/ml) increased 8.2- and 20.5-fold, respectively, of IL-6 secretion. However, 1 μM celastrol attenuated the activation of both PMA and TNFα on IL-6 secretion of PC-3 cells (Figure 3B).

**Figure 4:**
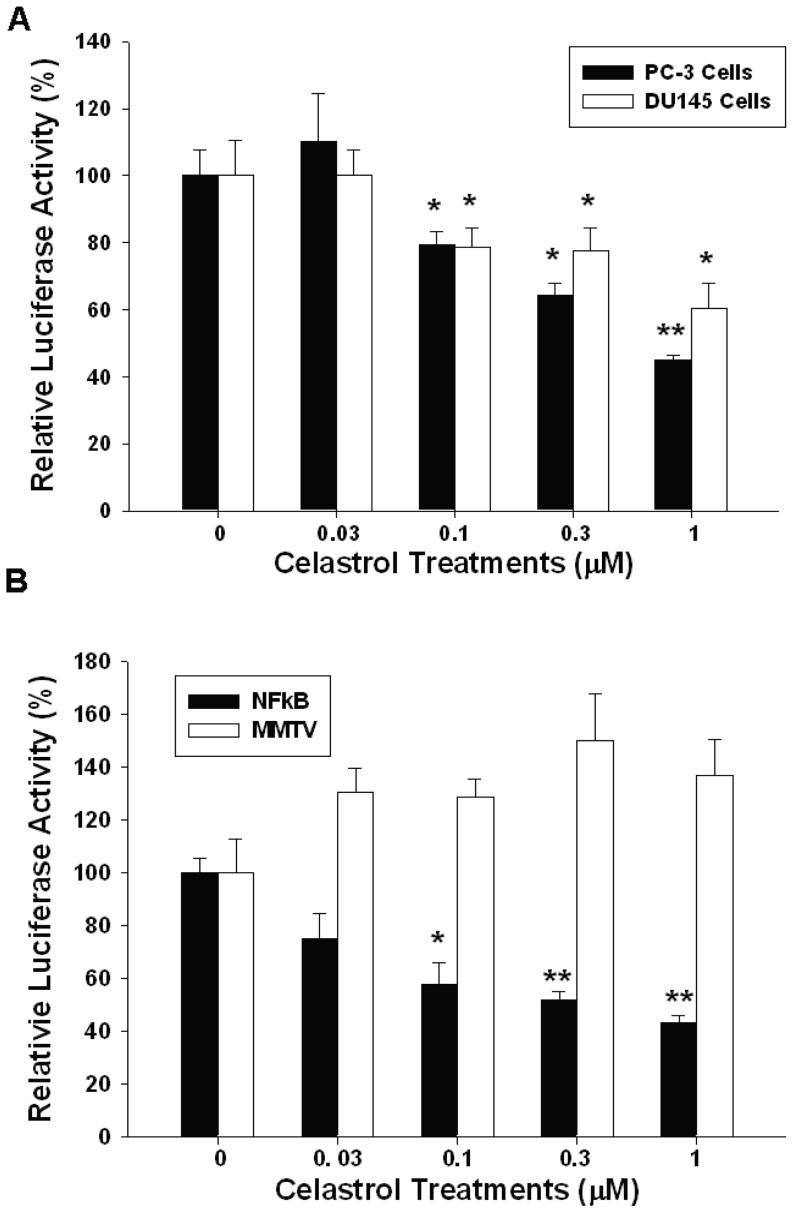
Celastrol downregulates interleukin-6 and NF-κB reporter activity in PC-3 cells. (A) Luciferase activity of IL-6 reporter vector (pIL6-SX)-transfected PC-3 (black bars) and DU145 (white bars) cells treated with different concentrations of celastrol as indicated. (B) Luciferase activity of NFκB reporter vectors (black bars)- and MMTV reporter vector (white bars)-transfected PC-3 cells treated with different concentrations of celastrol as indicated. Data are presented as the mean percentage ± SE (n = 6) of the reporter activities induced by celastrol treatments in relation to the control solvent-treated group. (*p<0.05; **p<0.01).

Transient gene expression assays using the human IL-6 reporter vector showed similar results. 1 μM celastrol treatment blocked 55% and 40%, respectively, of IL-6 promoter activity in PC-3 and DU145 cells (Figure 4A). In order to evaluate the effect of celastrol on the NF-κB activity, we conducted the transient gene expression assays using the NF-κB specific reporter vector containing four NF-κB response elements. Our results indicated that celastrol blocked NF-κB activity in PC-3 cells in a dosage-dependent manner (Figure 4B) but did not affect the promoter activity of the MMTV reporter vector which was derived by the promoter of mouse mammary tumor virus.

**Figure 5:**
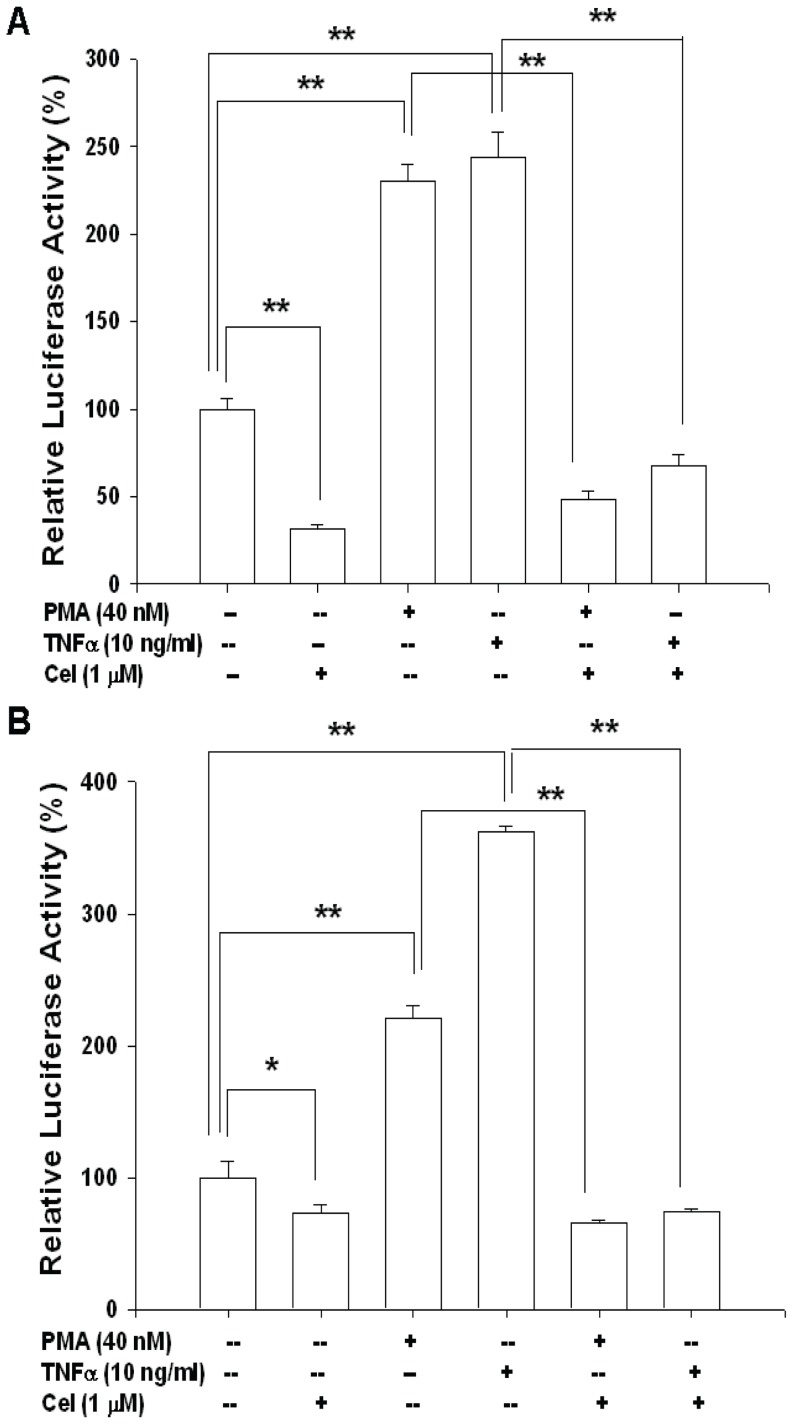
Celastrol blocks the activation of TNFα and PMA on interleukin-6 and NF-κB promoter activity. Luciferase activity of IL-6 reporter vector- (A) and NF-κB reporter vectors- (B) transfected PC-3 cells treated with 1 μM celastrol (Cel), 40 nM PMA, or/and 10 ng/ml TNFα. Data are presented as the mean percentage ± SE (n = 6) of the reporter activities induce by different treatments in relation to the control solvent-treated group. (*p<0.05; **p<0.01).

**Figure 6:**
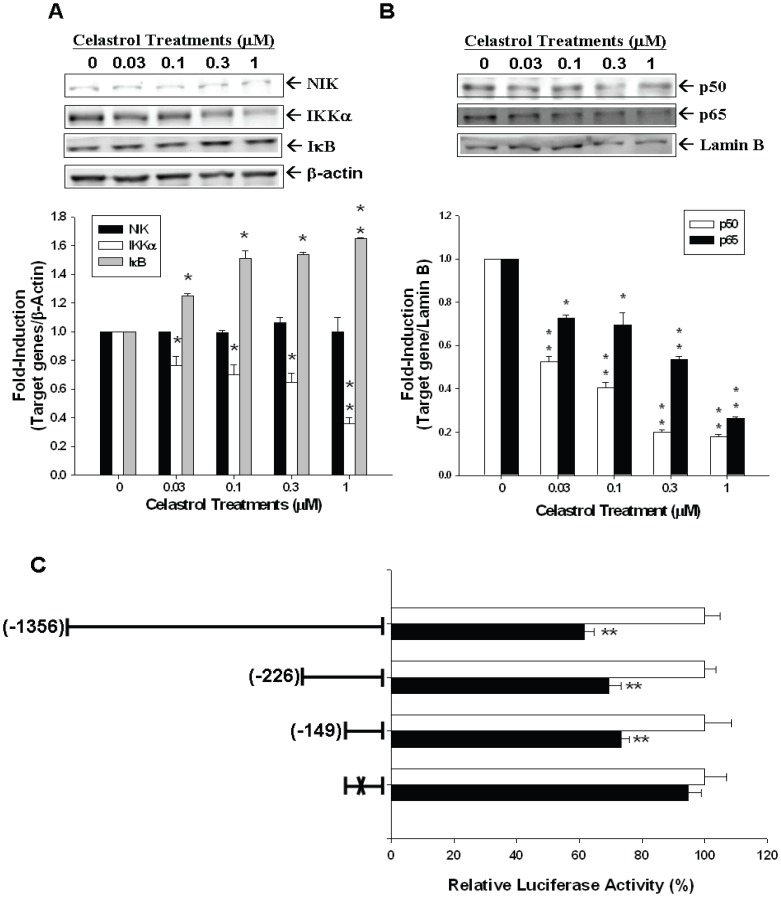
Celastrol represses PC-3 interleukin-6 expression through NF-κB signal pathway. PC-3 cells were treated with various concentrations of celastrol as indicated for 24 hours. The expressions of IKKα, NIK, IκB, and β-actin were determined in the cytoplasmic fraction (A), and the NFκBp50, NFκBp65, and Lamin B were determined in nuclear fraction (B) by immunoblotting assay (top). The quantitative analysis was done by determining the intensity of each band from three independent experiments (bottom). Data are presented as the fold-induction (± SE; n = 3) of the relative density of the target gene/β-actin (for IKKα, IκB, and NIK) and target gene/Lamin B (for NFκBp50 and NFκBp65) of treatments in relation to the control solvent-treated group (*p<0.05, **p<0.01). (C) Luciferase activity of nested deletion or mutation constructs of IL-6 reporter vectors-transfected PC-3 cells after treatment with control solvent (white bars) or 1 μM of celastrol (black bars). Data are presented as the mean percentage ± SE (n = 6) of the IL-6 reporter activity induced by celastrol treatment in relation to the control solvent-treated group. (“X” represents the mutant NF-κB response element; **P<0.01).

Further transient gene expression assay also indicated the PMA and TNFα enhanced the promoter activity of IL-6 (Figure 5A) and NF-κB (Figure 5B) reporter vectors in PC-3 cells, while these effects were blocked by celastrol. Immunblot assays revealed that celastrol treatments not only decreased the expression of IKKα in the cytoplasm but also the p50 and p65 in the nucleus of PC-3 cells. However, celastrol did not affect the expression of NF-kappa-B-inducing kinase (NIK) but enhanced the protein levels of IκB (Figure 6A and 6B). The results from the 5’-deletion reporter assays indicated the response element for the effects of celastrol on IL-6 promoter activity was located at −149 to +8 of the 5’-flanking of the human IL-6 gene (Figure 6C). Further transient gene expression assay indicated that celastrol did not affect the promoter activity of the mutant IL-6 reporter vector, in which the NF-κB binding site was mutated from AAATGTGGGATTTTTCCC to AAATGTTACATTTTCCC by site-directed mutagenesis (Figure 6C). Combined with the results shown in figure 5, we thus concluded that the effect of celastrol on IL-6 gene expression depends on the NFκB pathway (Figure 6C).

## Discussion

There are numerous studies showing positive effect of celastrol on cancer growth and metastasis in a variety of cancers, such as pancreatic cancer, lung cancer, and breast cancer [22]. In terms of prostate cancer, celastrol has been demonstrated to repress prostate cancer cell proliferation and induce apoptosis with downregulation of androgen receptor expression. Moreover, celastrol has also been found to exert antitumor effect on prostate cancer *in vivo* without obvious side effect [8], [14], [15]. Thus, application of celastrol to treat prostate cancer seems to be a promising alternative regimen. In this current study, we demonstrated that celastrol repressed PC-3 cell growth dose-dependently (Figure 1A) through cell cycle arrest at G0/G1 phase as indicated by increased G0/G1 phase cells (lower dose, Figure 1D) and apoptosis induction as indicated by increasing c-PARP expression, which is also supported by the result of tunnel assay (higher dose, Figure 1B, 1C), in line with previous reports [8], [14], [15].

IL-6, a multifunctional cytokine, has been shown to play a vital role in lots of important biologic activities in a cell- or tissue- specific manner and to be produced by a variety of cells, including cancer cells [10]. IL-6 belongs to a cytokine family comprising of IL-11, oncostain M, cardiotropin-1, etc [10]. IL-6 exerts its function through binding with a cell surface type 1 cytokine receptor complex which contains two components, i.e. the ligant-binding component (CD126) and the signal-transducing component (CD130). IL-6 has deemed as a growth factor through activation of JAK-STAT3, RAS, MAPK, Cox-2, PI3K/AKT, and Wnt pathways [10], [23]–[25]. Most cancers have been found to overexpress IL-6 and have an aberrant IL-6 signaling pathway [26]–[28]. Moreover, ample clinical studies have implicated that higher serum IL-6 concentrations in cancer patients are associated with advanced tumor stages and poor survival. Thus, blocking IL-6 signaling seems to be a rational direction to repress cancer growth [10]. Regarding prostate cancer, IL-6 expression is detectable in both epithelium and stroma of human prostates, with increased IL-6 expression in epithelium as the prostate tissues are getting transformation toward malignancy [29]. It has been shown that IL-6 is a growth factor for most prostate cancer cells and anti-IL-6 monoclonal antibody has been proven to effectively inhibit xenografted prostate cancer cells growth [30]. In addition, serum IL-6 level has been deemed as a prognostic marker in metastatic hormone-refractory prostate cancer patients [31]. In this study, we demonstrated for the first time that celastrol-mediated antitumor effect on PC-3 cells is IL-6-dependently as knockdown of IL-6 blunted the anti-proliferative effect of celastrol on PC-3 cells (Figure 2). Since IL-6 could be produced by PC-3 cells and acts in an autocrine or paracrine manner to stimulate cancer growth, we next measured whether the secretion of IL-6 by PC-3 cells is affected by celastrol treatment. As shown in figure 3A, a dose-dependent manner of downregulation of IL-6 secretion in PC-3 and DU145 cells by celastrol was observed as measured by ELISA. In addition, PMA- and TNFα-induced IL-6 secretion was also blocked by celastrol in PC-3 cells (Figure 3B). Transient gene reporter assay showed the similar result indicating that celastrol repressed IL-6 gene promoter activity in PC-3 and DU145 cells (Figure 4A). Collectively, we concluded that celastrol repressed IL-6 gene expression and secretion and inhibited prostate carcinoma cell growth IL-6-dependently.

The IKK/NF-κB signaling is an important pathway with aberrant NF-κB regulation existing in a myriad of cancers [32]–[34]. The NF-κB protein family comprises RelA (p65), RelB, c-Rel, p50 (p105 precursor), and p52 (p100 precursor) [33]. In the latent state, NF-κBs are bound to their inhibitor IκB (inhibitor of NF-κB) proteins and, thus, sequestered in the cytosol. Once receiving stimulation, IKK (IκB kinase), consisting of IKKα, IKKβ (two catalytic subunits), and NEMO/IKKγ (regulatory subunit), is activated to phosphorylate IκB, which, in turn, leads to proteasomal degradation of phosphorylated IκB and the release of NF-κB with subsequent nuclear translocation for gene expression modulation [35]. There are some transcriptional factors, such as AP-1, CCAAT enhancer binding protein, cAMP response element binding protein, as well as NF-κB reported to have potential binding sites within the human IL-6 gene promoter area and, thus, could interfere IL-6 gene expression in prostate cancer cells [21], [36]. NF-κB signaling pathway has also previously been shown to be one of the celastrol-targeted anticancer pathways [37]. As shown in Figure 4A and 4B, celastrol reduced the promoter activity of IL-6 reporter vector and NF-κB reporter vector, which contains the four repeats consensus NF-κB response elements, in PC-3 cells. PMA and TNFα both upregualted IL-6 and NF-κB promoter activity in PC-3 cells, however, this effect was blocked by celastrol (Figure 5A and 5B). Moreover, as determined by western blot assays, expression of IKKα in the cytoplasm and p50 and p65 in the nucleus of PC-3 cells were all inhibited by celastrol, while IκB expression was upregulated (Figure 6A, B). To the contrary, NIK , encoded by MAP3K14 gene in human and functioning as an alternative NF-κB pathway stimulator as binding with TRAF2 [38], was not repressed by celastrol treatment in PC-3 cells (Figure 6A and 6B), indicating celastrol seemed to repress NF-κB pathway directly in PC-3 cells. To further verify how celastrol regulates IL-6 gene expression in PC-3 cells, we conducted 5’-deletion reporter assays. As shown in figure 6C, our results indicated that the celastrol response element in IL-6 promoter area was located at −149 to +8 of the 5’-flanking of the human IL-6 gene, which also contains the NF-κB response element [21]. As we mutated NF-κB binding site from AAATGTGGGATTTTTCCC to AAATGTTACATTTTCCC by site-directed mutagenesis, the celastrol-mediated downregulation of IL-6 promoter activity was abolished. Taken together, based on our result, we concluded that celastrol inhibits IL-6 gene expression via the NF-κB pathway in PC-3 cells.

In conclusion, celastrol, one kind of active compound extracted from Chinese herbal, possesses potent anti-growth effect on prostate cancer through cell cycle arrest at G0/G1 and apoptosis induction. The growth inhibition of celastrol against prostate carcinoma cells depends on IL-6 pathway since knockdown of IL-6 blunts the growth inhibition induced by celastrol. Further, celastrol represses IL-6 gene expression and secretion in prostate carcinoma cells via the NF-κB signaling pathway.
